# Early initiation of eculizumab treatment in patients with atypical haemolytic uraemic syndrome improves long-term outcomes: a pooled analysis of clinical trials

**DOI:** 10.1186/cc14410

**Published:** 2015-03-16

**Authors:** J Vande Walle, Y Delmas, G Ardissino, J Wang, J Kincaid, H Haller

**Affiliations:** 1 University Hospital Ghent, Belgium; 2 Centre Hospitalier Universitaire de Bordeaux, France; 3 Ospedale Maggiore Policlinico, Milan, Italy; 4 Alexion Pharmaceuticals, Cheshire, CT, USA; 5 Medical School Hannover, Germany

## Introduction

Atypical haemolytic uraemic syndrome (aHUS) is a severe, life-threatening disease requiring rapid treatment to inhibit complement-mediated thrombotic microangiopathy (TMA) and avoid irreversible organ damage. Four prospective clinical trials have reported the safety and efficacy of eculizumab (Ecu) in the treatment of aHUS [1,2]. We report data from a pooled analysis of these trials on renal function in patients starting Ecu within ≤7 days or >7 days after the current aHUS manifestation.

## Methods

Data from four phase 2, open-label, single-arm trials including both paediatric and adult patients with aHUS were pooled. Patients with a documented date of onset of current TMA manifestation and a baseline estimated glomerular filtration rate (eGFR) of <90 ml/ minute/1.73 m^2^ were included. Changes from baseline in eGFR were analysed at study visits using a one-sample *t *test.

## Results

**Figure 1:**
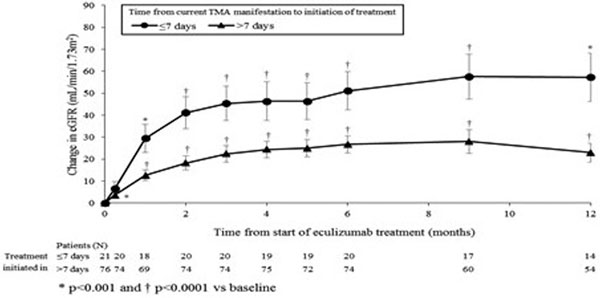
**Mean change in eGFR from baseline over 1 year (standard error)**.

Data from 97 patients were analysed: median (range) age at enrolment was 29 (0 to 80) years; 62% of patients were females; median (range) duration of current manifestation to start of Ecu treatment was 23 (1 to 1,447) days; median (range) baseline eGFR was 15.9 (5.6 to 76.1) ml/minute/1.73 m^2^. Ecu treatment was started in 21 patients in ≤7 days and 76 patients in >7 days after presentation with TMA. Median eGFR was 11 ml/minute/1.73 m^2^ for the patients started within 7 days and 16 ml/minute/1.73 m^2^ for those initiating >7 days. The mean change from baseline in eGFR for patients starting Ecu in ≤7 days and in >7 days after presentation with TMA were 57 and 23 ml/minute/1.73 m^2 ^at 1 year, respectively (Figure 1).

## Conclusion

This pooled analysis indicates that patients treated with Ecu within 7 days of a TMA manifestation had a greater improvement in eGFR over time than patients in whom treatment was delayed. These data show the importance of rapid diagnosis and treatment of aHUS for recovery of renal function.

## References

[B1] Legendre C N Engl J Med 2013 368 2169 81 10.1056/NEJMoa1208981 23738544

[B2] Keating GM Drugs 2013 73 2053 66 10.1007/s40265-013-0147-7 24249647

